# Wnt/β-Catenin Pathway Activation Confers Fumonisin B1 Tolerance in Chicken Intestinal Organoid Monolayers by Enhancing Intestinal Stem Cell Function

**DOI:** 10.3390/ani15192850

**Published:** 2025-09-29

**Authors:** Shuai Zhang, Yanan Cao, Yiyi Shan, Xueli Zhang, Liangxing Xia, Haifei Wang, Shenglong Wu, Wenbin Bao

**Affiliations:** College of Animal Science and Technology, Yangzhou University, Yangzhou 225009, China; shuai_zhang1990@163.com (S.Z.); yncao1994@163.com (Y.C.); mz120211434@stu.yzu.edu.cn (Y.S.); zhangxueli1632022@163.com (X.Z.); lxxia0830@163.com (L.X.); hyfiwang@yzu.edu.cn (H.W.)

**Keywords:** chicken, fumonisin B1, intestinal organoid, barrier function, Wnt/β-catenin signaling pathway

## Abstract

**Simple Summary:**

Fumonisin B1 (FB1), a common mycotoxin in moldy grains/feeds, harms livestock and threatens food safety, but poultry’s FB1 tolerance mechanism remains unclear. Traditional 3D chicken organoid models cannot simulate epithelial monolayer–toxin interaction, limiting FB1-intestinal crosstalk studies. This study used a 2D chicken intestinal organoid monolayer model to explore poultry’s FB1 resistance mechanisms. Unlike porcine models, FB1 did not reduce the monolayer’s transepithelial electrical resistance, disrupt tight junction gene expression, or fluctuate inflammatory factor levels in chickens. Mechanistically, FB1 activates the Wnt/β-catenin pathway to enhance intestinal stem cell function and promote epithelial regeneration, boosting chickens’ FB1 resistance. The findings reveal a FB1 tolerance strategy in poultry and provide new insights for mycotoxin control.

**Abstract:**

Fumonisin B1 (FB1) is a prevalent mycotoxin in moldy grains and feeds, highly toxic to livestock and compromising product quality while threatening food safety. Poultry exhibit low susceptibility to FB1, but the underlying tolerance mechanisms remain unclear. Traditional 3D chicken intestinal organoid models cannot simulate direct interaction between the epithelial monolayer and FB1, limiting the study of FB1–chicken intestinal crosstalk. Here, we established a 2D chicken intestinal organoid monolayer model, derived from intestinal crypts of 18-day-old specific pathogen-free chicken embryos, to systematically explore poultry’s resistance mechanisms against FB1. Using this model, we compared FB1-induced effects with those in a porcine intestinal epithelial cell model. Results showed that FB1 exposure did not reduce transepithelial electrical resistance, induce abnormal expression of tight junction genes, or cause significant fluctuations in inflammatory factor levels in chicken intestinal organoid monolayers. Mechanistically, FB1 enhances chicken intestinal stem cell function by activating the Wnt/β-catenin pathway, thereby promoting epithelial regeneration and renewal to increase FB1 resistance and decrease toxin sensitivity in chickens. This study reveals a strategy for enhancing FB1 tolerance in poultry by promoting intestinal stem cell function, providing a new perspective for developing mycotoxin prevention and control strategies.

## 1. Introduction

Mycotoxins are widespread in moldy grain and feed. Fumonisins are highly water-soluble and heat-stable mycotoxins primarily produced by Fusarium mold fungi, notably *Fusarium verticillioides* [1]. Fumonisins are categorized into A, B, C and P series, among which fumonisin B1 (FB1) is the most abundant [1,2] (accounting for almost 70% of total fumonisins) and toxic, primarily contaminating maize and its products [1,2,3,4]. Given maize’s high proportion in poultry feed, birds are prone to high dietary intake of FB1 [5]. Upon ingestion through contaminated feed, FB1 undergoes gastrointestinal absorption and bioaccumulates in intestinal tissues over time [6,7]. FB1 exerts multifaceted toxicity to livestock, including growth retardation, immunotoxicity, decreased production, reproductive toxicity and carcinogenicity (organ canceration), threatening animal and human health [8]. Intriguingly, species differ in sensitivity, with poultry showing relatively high tolerance to FB1 [6,9,10]. Although poultry show relative tolerance to FB1, the specific mechanism of tolerance remains unelucidated. As the scale of poultry farming continues to expand globally, FB1-contaminated poultry products pose a major challenge to poultry food safety. However, there is a lack of systematic research on the intestinal effects of FB1 in poultry, which is not commensurate with its status as a key food safety contaminant under global surveillance, and in-depth mechanistic studies are urgently needed.

The gastrointestinal tract is chronically exposed to a wide range of exogenous substances, including microbes and toxic compounds [11], and serves as a major barrier against intestinal ingested toxins such as FB1 [12]. The intestinal epithelium, composed of a monolayer of different cell types, serves as a crucial barrier that maintains the balance between the host and the intestinal microenvironment. To cope with external and internal stress, the epithelium undergoes continuous renewal, replenishing damaged epithelial cells in the intestinal mucosa to maintain its integrity [13]. Intestinal epithelial homeostasis is maintained by the continuous proliferation and differentiation of stem cells in the crypts [14,15], which give rise to transit-amplifying (TA) daughter cells. TA cells can differentiate into various mature epithelial cell types such as absorptive cells, goblet cells, Paneth cells, tuft cells and enteroendocrine cells [16,17]. Together, these highly specialized different types of intestinal epithelial cells (IECs) are firmly connected by a complex network of tight junctions, adherens junctions and gap junction proteins [18]. Studies have shown that impairment of epithelial barrier function due to food-derived mycotoxins may be one of the most important causes of induced intestinal pathology [19]. Various environmental factors including pathogens (e.g., *Salmonella*), probiotics (e.g., *Lactobacillus reuteri*) and mycotoxins (e.g., zearalenone) have been shown to affect epithelial repair and homeostasis by modulating the Wnt/β-catenin signaling pathway [20,21,22]. This pathway is critical in the continued expansion of intestinal stem cells (ISCs) and maintenance of intestinal epithelial renewal, and is one of the factors determining the fate of ISCs during repair after injury [23]. Upon Wnt ligand binding to the Frizzled-lipoprotein receptor-related protein (LRP) transmembrane receptor, this pathway inhibits β-catenin degradation. Accumulated β-catenin translocates to the nucleus and forms a transcriptional complex with TCF/LEF factors to further induce the transcription of target genes [24].

Although FB1’s cytotoxicity is well documented, its effect on ISC self-renewal remains elusive, partly due to the lack of suitable chicken intestinal models. Intestinal organoids, self-organizing in vitro epithelial structures, recapitulate native intestinal morphology and function [25], and intestinal organoids have been established using chicken embryos at 18–20 days of development [26,27,28]. In this three-dimensional (3D) model, intestinal epithelial cells are highly polarized with a lumen-facing apical membrane (brush border) and a basal membrane in contact with the underlying extracellular matrix. This structural arrangement hinders direct access to toxins or nutrients, and while “apical-out” mammalian models have been developed to address this limitation, challenges such as incomplete polarization reversal (~20% failure rate) and declining viability over time significantly restrict their experimental utility [29]. This has driven interest in two-dimensional (2D) models of the intestinal epithelium, which allow for direct manipulation and access to the apical surface of the epithelium. However, there is a lack of quality two-dimensional chicken intestinal models with confluent monolayer structure and complete barrier function, and this technological bottleneck has severely restricted studies on toxin–epithelium interactions.

Here, we established a novel 2D chicken intestinal organoid system with a confluent, barrier-intact monolayer. Using this model, we systematically investigated the impacts of FB1 on chicken intestinal barrier function, inflammatory responses, ISC self-renewal, and Wnt/β-catenin pathway. Our research provides a reliable platform for mycotoxin–epithelium interaction studies and elucidates how poultry enhance FB1 tolerance via ISC function, offering new insights into mycotoxin control.

## 2. Materials and Methods

### 2.1. Cell Viability Assay

FB1 cytotoxicity in IPEC-J2 cells was assessed using the CCK-8 kit (Yeasen, Shanghai, China), according to the manufacturer’s instructions. Specifically, IPEC-J2 cells were seeded at 3 × 10^4^ cells per well in 96-well plates and incubated for 12 h. Different concentrations of FB1 were added to each well and incubation continued for 24 h. After adding 10 μL CCK-8 reagent for 2 h, absorbance at 450 nm was quantified using a Tecan Spark 10 M microplate reader (Tecan, Männedorf, Switzerland).

### 2.2. Isolation and Culture of Chicken Intestinal Organoids

Intestinal crypts were first isolated from the small intestine of 18-day-old specific pathogen-free (SPF) chicken embryos. The freshly excised intestine was cut open longitudinally, rinsed with phosphate-buffered saline (PBS) to remove luminal contents, then cut transversely into 5 mm segments. These segments were digested in 5 mM EDTA for 30 min on ice; after dissociation, crypts were washed three times with cold PBS. Isolated crypts were counted and embedded in Matrigel matrix (Corning, NY, USA) with IntestiCult™ Organoid Growth Medium (StemCell Technologies, Vancouver, BC, Canada) at 37 °C with 5% CO_2_, and fresh medium was replaced every 48–72 h following established protocols [30,31]. The organoid morphology was monitored daily under a light microscope until well developed.

### 2.3. Hematoxylin-Eosin Staining of Chicken Intestinal Organoids

The well-developed chicken intestinal organoids were harvested and fixed with 4% paraformaldehyde at 4 °C for 1 h. Cells were centrifuged into a pellet, re-suspended in cold 30% sucrose solution and incubated at 4 °C for 3 h. Organoids were repelleted and resuspended in approximately 50 μL of the remaining supernatant, and then overlaid on 450 μL of O.C.T. embedding medium within a Tissue-Tek cryomold (Sakura, CA, USA). The organoids were centrifuged to sediment at the bottom of the embedding mold, followed by freezing and preparation for cryosectioning. Hematoxylin and eosin (H&E) staining of frozen sections was performed using an H&E Stain Kit (Solarbio, Beijing, China).

### 2.4. Indirect Immunofluorescence

The intestinal organoids and organoid monolayers were fixed with 4% paraformaldehyde for 20 min at 37 °C, permeabilized with 0.1% Triton X-100 in PBS for 10 min, blocked with 5% bovine serum albumin (BSA) for 1 h at 37 °C, and incubated overnight at 4 °C with primary antibody at 1:100 or 1:200 [enterocyte marker: anti-Villin (ab130751, Abcam, Cambridge, UK) and anti-E-cadherin (610181, BD Biosciences, San Jose, CA, USA), TA cell marker: anti-Ki67 (9449S, Cell Signaling Technology, Danvers, MA, USA), Paneth cell marker: anti-Lyz (sc-27958, Santa Cruz Biotechnology, Dallas, TX, USA), or intestinal stem cell marker: anti-Lgr5 (NBP1-28904, Novus Biologicals, Centennial, CO, USA)]. After washing, an appropriate second antibody (Dylight 488, 549, or 649 goat anti-mouse IgG H&L, 1:200 dilution) was applied for 30 min, followed by a 10 min incubation with 1 μg/mL DAPI at room temperature to stain nuclei. Fluorescent images were captured using a Leica TCS SP8 STED laser scanning confocal microscope (Leica, Mannheim, Germany) and analyzed with Leica Application Suite X (version 3.4.2) software.

### 2.5. Development of 2D Chicken Intestinal Organoid Monolayer

Mature chicken enteroids were employed to generate planar (two-dimensional) monolayers. Organoids were retrieved from Matrigel after two washes with ice-cold DMEM/F12 medium to remove residual base matrix, then dissociated using TrypLE^TM^ Express (Gibco, Carlsbad, CA, USA) at 37 °C for 10 min, with gentle pipetting every 3 min to ensure complete dissociation. The dissociated cells were plated into 48-well plates (Corning, NY, USA) or Transwell culture plates (Corning, NY, USA) and maintained in a humidified 5% CO_2_ incubator at 37 °C. To monitor monolayer formation, confluency was assessed daily via light microscopy. Cells were observed to proliferate gradually and form intercellular connections, and confluent intestinal organoid monolayers were formed within 3–4 days. Once confluent, the monolayers were used for subsequent experiments.

### 2.6. Scanning Electron Microscopy

For scanning electron microscopy (SEM), chicken intestinal organoid monolayers (seeded in 24-well plates) were fixed with 2.5% glutaraldehyde at 4 °C for 2 h, washed three times with PBS, and dehydrated through a graded ethanol series (30%, 50%, 70%, 80%, 90%, and 100%, 10 min each). After freeze-drying, the samples were sputter coated with gold and imaged with a GeminiSEM 300 scanning electron microscope (Carl Zeiss, Oberkochen, Germany).

### 2.7. EdU Fluorescence Assay

EdU (5-ethynyl-2′-deoxyuridine) is a thymidine analogue that can be specifically incorporated into the DNA of cells during DNA replication. For the EdU fluorescence assay, chicken intestinal organoid monolayers were first seeded in 24-well plates, and cell proliferation was then assessed using the Cell-Light EdU Apollo488 In Vitro Kit (RiboBio, Guangzhou, China) strictly following the manufacturer’s instructions. Specifically, EdU was added to the organoid culture medium at the recommended final concentration and incubated at 37 °C with 5% CO_2_ for 4 h to ensure sufficient incorporation into replicating DNA. After incubation, organoid monolayers were fixed with 4% paraformaldehyde at room temperature for 10 min to preserve cell structure, then permeabilized with 0.5% Triton X-100 in PBS for 15 min to facilitate dye penetration. Next, the monolayers were stained with the kit’s Apollo dye solution for 30 min in the dark to label proliferating cells. Finally, cellular nucleic acids were counterstained with DAPI for 10 min to visualize cell nuclei. Fluorescent images were captured with a Leica TCS SP8 STED laser scanning confocal microscope (Leica, Mannheim, Germany) and analyzed with Leica Application Suite X (version 3.4.2) software.

### 2.8. Transepithelial Electrical Resistance

Mature chicken intestinal organoids were dissociated into single cells and plated on 24-well transparent Transwell inserts (0.4 μm pore size; 6.5 mm diameter; Corning, NY, USA). The transepithelial electrical resistance (TEER) of monolayers with or without FB1 exposure at indicated time points was monitored with Millicell ERS-2 (Merck Millipore, Burlington, MA, USA). Resistance of a unit area = (Total resistance − blank resistance) (Ω)  ×  Effective membrane area (cm^2^).

### 2.9. RNA Extraction and RT-qPCR

Total RNA was extracted from collected samples with RNAiso reagent (TaKaRa, Dalian, China). The quantity and purity of the extracted RNA were quantified with a Nanodrop spectrophotometer (Thermo Scientific, Waltham, MA, USA). Subsequently, 1 μg of RNA from each sample was reverse transcribed into cDNA using the HiScript II 1st Strand cDNA Synthesis Kit (Vazyme, Nanjing, China). Quantitative real-time PCR (RT-qPCR) was conducted with SuperStar Universal SYBR Master Mix (CWBIO, CW3360M, Taizhou, China) on an Applied Biosystems 7500 real-time PCR system (Applied Biosystems, Foster City, CA, USA). *GAPDH* served as the reference gene, and relative gene expression levels were analyzed using the 2^−△△CT^ method across three biological replicates. Primers were designed via Primer-BLAST (https://www.ncbi.nlm.nih.gov/tools/primer-blast/index.cgi (accessed on 16 September 2024)) and validated via melt curve analysis to ensure specificity and efficiency. Primer sequences for target genes are provided in Table S1.

### 2.10. Statistical Analysis

Data are presented as the mean ± standard deviation (SD) from three independent experiments. Prior to analysis, outliers were identified via Grubbs’ test with significance level alpha = 0.05 and excluded from subsequent analyses. For comparisons between two groups, Student’s *t*-test was performed using GraphPad Prism 6 software. Statistical difference was defined as follows: ns *p*  >  0.05 no significant difference, * *p*  <  0.05 significant, ** *p*  <  0.01 highly significant.

## 3. Results

### 3.1. Isolation and Identification of Chicken 3D Intestinal Organoids

**Figure 1:**
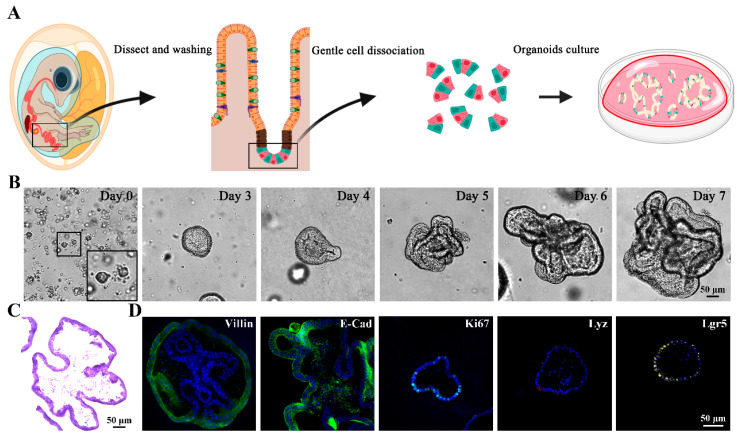
Isolation and identification of chicken 3D intestinal organoids. (**A**) Schematic illustration of the isolation and culture protocol for chicken intestinal organoids. (**B**) Light microscopic images showing the time-course development of 3D chicken intestinal organoids. Scale bar = 50 μm. (**C**) H&E staining of 3D chicken intestinal organoids. Scale bar = 50 μm. (**D**) Immunofluorescence assays (IFA) of 3D chicken intestinal organoids for enterocytes (Villin and E-Cad, green), proliferating cells (Ki67, green), Paneth cells (Lyz, red) and intestinal stem cells (Lgr5, yellow). Nuclei were stained with DAPI (blue). Scale bar = 50 μm.

Figure 1A outlines the isolation and culture process of intestinal crypts from 18-day-old chicken embryonic intestinal tissue (Figure 1A). After digestion, crypt cells were isolated and embedded in Matrigel for 7 days to generate mature organoids. During the incubation process, the spherical organoids gradually developed the emergent form with mature villi and crypt-like structures, and further formed more complex multilobular structures with prolonged incubation times (Figure 1B). HE staining of frozen section of chicken intestinal organoids revealed luminal structures (Figure 1C). To further characterize the chicken 3D intestinal organoids, immunofluorescence staining (IFA) was performed to identify the cell types existing in the intestinal organoids. Enterocytes were identified by the expression of Villin and E-cadherin, transit-amplifying cells were stained for proliferation marker Ki67, Paneth cells were detected through lysozyme expression, and stem cells were confirmed by positive Lgr5 staining (Figure 1D). These findings demonstrate that the cultured intestinal organoids comprise multiple cell types, partially recapitulating the cellular complexity of the chicken intestinal epithelium.

### 3.2. Establishment and Characterization of Chicken Intestinal Organoid Monolayers

**Figure 2:**
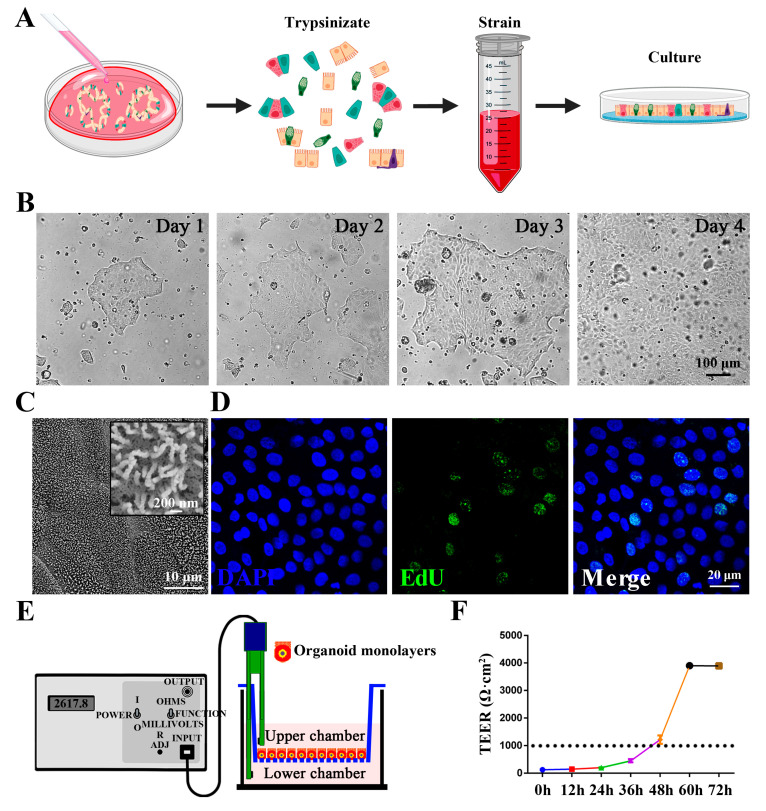
Establishment and characterization of chicken intestinal organoid monolayers. (**A**) Schematic illustration of chicken 2D intestinal organoid monolayer establishment. (**B**) The dissociated chicken organoids were regenerated into a confluent intestinal organoid monolayer within 4 days. Scale bar = 100 μm. (**C**) The intestinal microvilli morphology of confluent intestinal organoid monolayers as observed by SEM. Scale bars = 200 nm or 10 μm. (**D**) Confocal micrographs showing nuclear staining (DAPI, blue) and EdU staining (green) in chicken intestinal organoid monolayers. Scale bar = 20 μm. (**E**) Schematic of TEER measurement across organoid monolayers in transwell plates. (**F**) TEER dynamics of chicken organoid monolayers over 48 h.

Generally, most harmful substances and intestinal pathogens invade the intestines via the apical surface of epithelial cells [32]. However, the internal lumen of 3D organoids inherently limits direct access of exogenous substances to invade the organoid through the apical epithelial surface. Therefore, we developed chicken 2D intestinal organoid monolayers by dissociating chicken 3D intestinal organoids into single cells (Figure 2A). Within 24 h, epithelial islands formed and expanded into a confluent epithelial monolayer by day 4 (Figure 2B). Electron microscopy analysis revealed that these monolayers retained key features of intestinal epithelial cells, including abundant microvilli on the apical surface (Figure 2C). EdU staining confirmed proliferative activity in a subset of monolayer cells (Figure 2D), demonstrating their capacity for self-renewal. To further assess the barrier function of these monolayers, we measured transepithelial electrical resistance (TEER), a gold-standard metric for tight junction integrity and barrier function [33]. Chicken intestinal organoid monolayers formed a robust barrier with TEER values exceeding 1000 Ω·cm^2^ (Figure 2E,F), confirming their functional integrity. Collectively, these results indicate that the chicken intestinal organoid monolayers recapitulate both the cellular diversity and the barrier functions of the primary chicken intestinal epithelium.

### 3.3. Effect of FB1 on the Tight Junction Function and Inflammatory Response in Chicken Intestinal Organoid Monolayers

**Figure 3:**
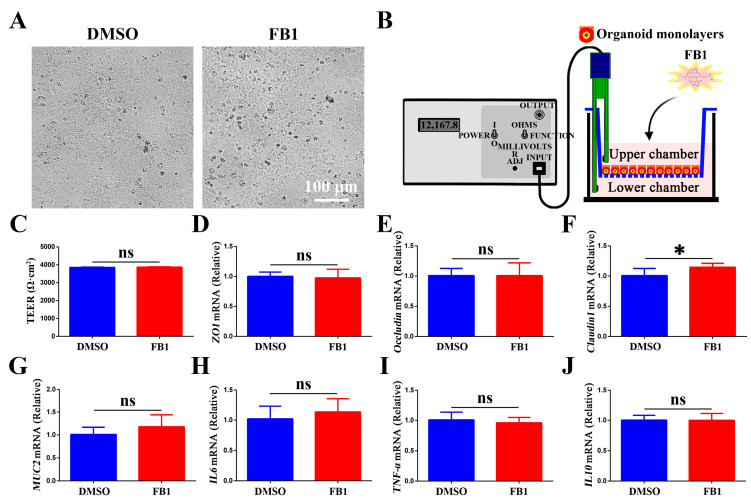
Effects of FB1 on the tight junction function and inflammatory response in chicken intestinal organoid monolayers. (**A**) Light microscopic images of chicken intestinal organoid monolayers treated with FB1 for 48 h. Scale bar = 100 μm. (**B**,**C**) TEER values of chicken intestinal organoid monolayers treated with FB1 for 48 h. (**D**–**G**) mRNA levels of tight junction proteins (*ZO1*, *Occludin*, and *Claudin1*) and goblet cell secreted mucin (*MUC2*) in FB1-treated chicken intestinal organoid monolayers at 48 h. (**H**–**J**) mRNA levels of pro- and anti-inflammatory cytokines *IL6*, *TNF-α*, and *IL10* from FB1-treated chicken intestinal organoid monolayers at 48 h. * *p* < 0.05, ns: not significant.

Subsequently, we evaluated the effect of 20 μg/mL FB1 on chicken intestinal organoid monolayers. Microscopic observation revealed no obvious morphological changes in FB1-exposed chicken intestinal organoid monolayers (Figure 3A). A central structural basis for intestinal barrier function, the intestinal epithelial tight junction (TJ) plays a critical role, and damage to TJs typically leads to elevated intestinal permeability [34]. Unexpectedly, FB1 exposure did not significantly reduce TEER values compared to controls (Figure 3B,C). Additionally, RT-qPCR analysis of tight junction-associated gene expression showed that FB1 treatment did not significantly affect *ZO1* and *Occludin* expression, but increased *claudin1* levels (Figure 3D–F). Similarly, mRNA levels of goblet cell secreted mucin (*MUC2*) remained unchanged in FB1-treated chicken intestinal organoid monolayers (Figure 3G). In contrast, the same FB1 concentration exerted cytotoxic effects on porcine intestinal epithelial cells and impaired their barrier function, as evidenced by decreased mRNA levels of *ZO1*, *Occludin*, *claudin1*, and *MUC2* (Figure S1A–E). As well, FB1 promoted the expression of pro-inflammatory factors interleukin 6 (*IL6*) and tumor necrosis factor-α (*TNF-α*), and inhibited anti-inflammatory factor interleukin 10 (*IL10*), in porcine intestinal epithelial cells (Figure S1F–H). Notably, the expression of pro-inflammatory factors *IL6* and *TNF-α* and anti-inflammatory factor *IL10* did not change significantly in FB1-treated chicken intestinal organoid monolayers (Figure 3H–J), suggesting that FB1 did not directly induce inflammatory responses in chicken intestinal organoid monolayers. These findings collectively suggest that 20 μg/mL FB1 had no significant effect on the integrity of the intestinal barrier and inflammatory response in chickens, but it had a clear barrier-damaging and pro-inflammatory effect on porcine intestinal epithelial cells, suggesting that there are species differences in the intestinal toxicity of FB1 and the sensitivity of chickens to FB1 was lower than that of pigs.

### 3.4. FB1 Activates the Canonical Wnt/β-Catenin Signaling in Chicken Intestinal Organoid Monolayers

**Figure 4:**
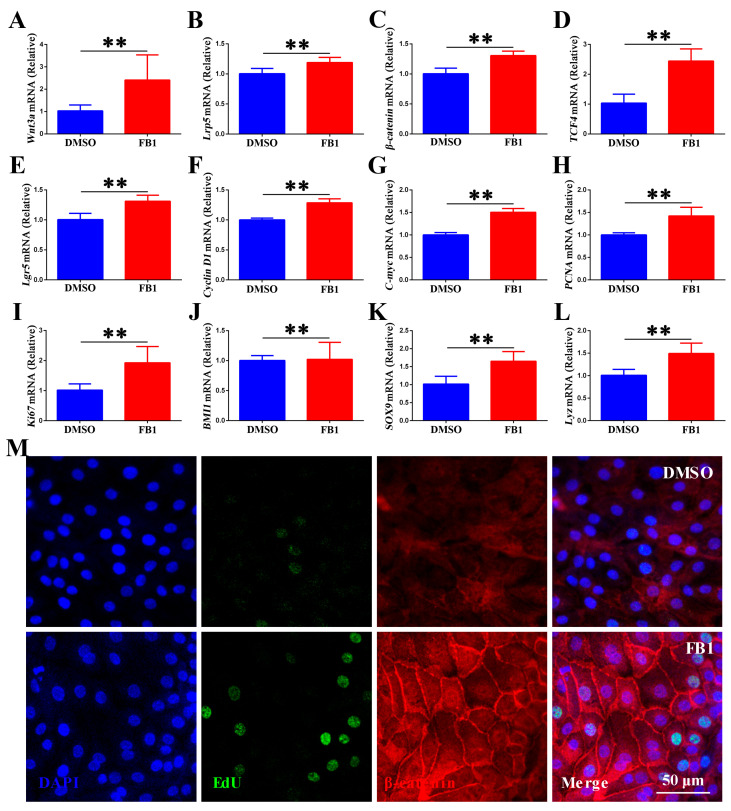
FB1 activates the Wnt/β-catenin signaling pathway in chicken intestinal organoid monolayers. 2D chicken intestinal organoids were treated with 20 μg/mL FB1 for 48 h. (**A**–**L**) mRNA levels of *Wnt3a*, *Lrp5*, *β-catenin*, *TCF4*, *Lgr5*, *Cyclin D1*, *c-myc*, *PCNA*, *Ki67*, *Bmi1*, *SOX9* and *Lyz* of chicken intestinal organoid monolayers. (**M**) Confocal microscopic images showing the expression and subcellular localization of β-catenin. Edu is stained green, β-catenin red, and nuclei blue. Scale bar = 50 μm. ** *p* < 0.01.

The intestinal epithelium undergoes continuous renewal to maintain tissue integrity [35]. This rapid turnover allows intestinal epithelial cells to quickly respond to environmental changes such as toxic stimuli (toxins), thereby preserving barrier integrity and functional adaptability. The renewal of ISCs driven by Wnt/β-catenin is crucial for maintaining intestinal homeostasis [36]. Given these above observations, we hypothesized that FB1 exposure activates ISCs to sustain the integrity of chicken intestinal organoid monolayers. To test this hypothesis, RT-qPCR was performed to measure the mRNA expression of critical components in the Wnt/β-catenin pathway. The data showed that FB1 exposure dramatically upregulated the expression of Wnt/β-catenin pathway genes (*Wnt3a*, *Lrp5*, *β-catenin*, and *TCF4*) and Wnt target genes (*Lgr5*, *Cyclin D1* and *c-myc*) (Figure 4A–G). The mRNA levels of *PCNA* (transiently amplifying (TA) cells), *Ki67* (proliferating cells), *BMI1* and *SOX9* (ISCs), and *Lyz* (Paneth cells) were also significantly upregulated (Figure 4H–L). IFA staining of chicken intestinal organoid monolayers revealed that FB1 induced cytosolic β-catenin accumulation and activated the canonical Wnt/β-catenin pathway via β-catenin nuclear translocation, thereby enhancing the proliferation of ISCs as indicated by increased EdU-positive cell counts (Figure 4M). These results indicate that FB1 activates the Wnt/β-catenin signaling pathway in chicken ISCs, thereby enhancing their self-renewal and proliferative capacity.

### 3.5. Wnt/β-Catenin Signaling Promotes FB1-Induced Renewal and Regeneration of Chicken Intestinal Epithelium In Vitro

**Figure 5:**
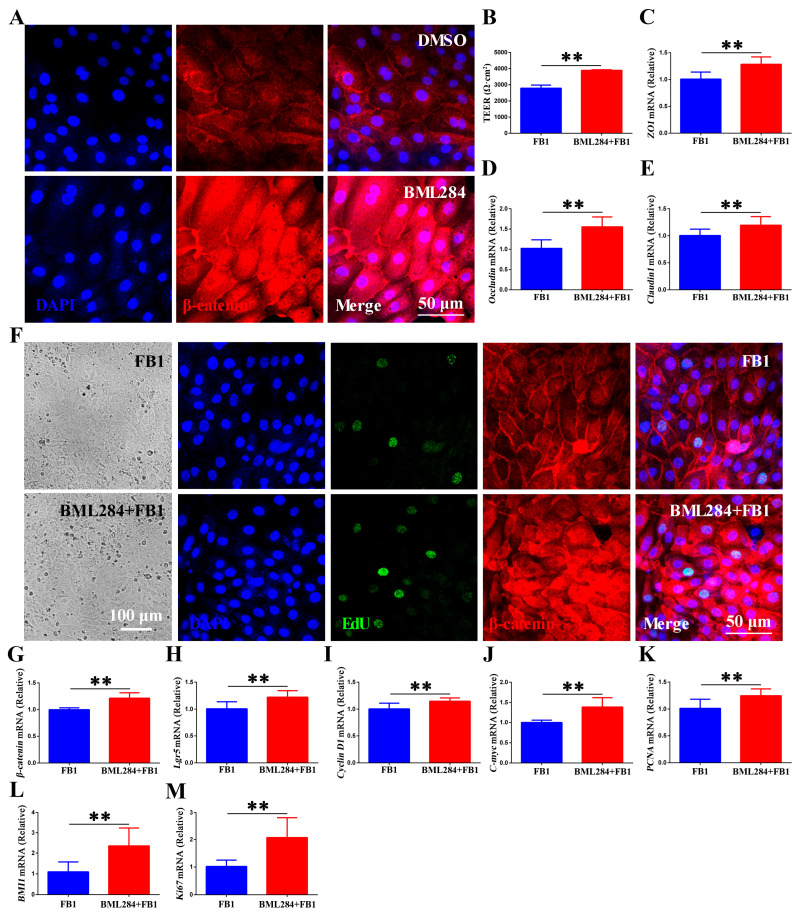
Wnt/β-catenin signaling promotes the renewal and regeneration of the chicken intestinal epithelium following FB1 exposure. (**A**) Confocal microscopic images showing β-catenin expression and localization in BML284-treated chicken intestinal organoid monolayers. Scale bar = 50 μm. (**B**–**M**) Chicken intestinal organoid monolayers were pretreated with 10 μM BML284 for 24 h and then incubated with 20 μg/mL FB1 for 48 h. (**B**) TEER values of chicken intestinal organoid monolayers. (**C**–**E**) mRNA levels of *ZO1*, *Occludin* and *Claudin1* of chicken intestinal organoid monolayers. (**F**) Cell morphology observed by light microscope (scale bar = 100 μm). Confocal images of nuclear staining (blue), EdU staining (green) and β-catenin staining (red) in chicken intestinal organoid monolayers. Scale bar = 50 μm. (**G**–**M**) mRNA levels of *β*-catenin, *Lgr5*, *Cyclin D1*, *c-myc*, *PCNA*, *BMI1*, and *Ki67* in chicken intestinal organoid monolayers. ** *p* < 0.01.

The above results suggest that FB1 exposure triggers activation of the Wnt/β-catenin signaling pathway in chicken organoid monolayers. To further confirm the function of this pathway in FB1-induced epithelial cell responses, we treated the monolayers with 10 μM BML284 (a small-molecule activator of the Wnt/β-catenin pathway). IFA results showed increased β-catenin levels with nuclear accumulation in BML284-treated chicken intestinal organoid monolayers (Figure 5A). We subsequently monitored TEER in FB1-exposed chicken intestinal organ monolayers after pretreatment with BML284 in a Transwell system. Unsurprisingly, BML284 further enhanced epithelial cell integrity, as evidenced by increased TEER values and significantly higher levels of tight junction-related genes (*ZO1*, *Occludin*, *Claudin1*) (Figure 5B–E). Importantly, we further investigated the role of Wnt/β-catenin in intestinal epithelial renewal and regeneration during FB1 exposure. BML284 had no effect on the morphology of cells but markedly increased the expression and translocation of β-catenin (Figure 5F,G). Simultaneously, it also increased mRNA levels of Wnt target genes (*Lgr5*, *Cyclin D1* and *c-myc*), ISC markers (*BMI1*), and proliferation-related genes (*PCNA* and *Ki67*) (Figure 5H–M). These findings demonstrate that Wnt/β-catenin signaling pathway enhances the self-renewal and regeneration of ISCs, accelerates the renewal and replenishment shedding of FB1-contaminated chicken intestinal epithelial cells, further strengthens the intestinal barrier function, and thus reduces the susceptibility of chickens to FB1.

## 4. Discussion

As poultry farming expands globally, food safety concerns stemming from mycotoxin contamination (especially FB1) have drawn increasing scrutiny. Notably, the widespread occurrence of FB1 in poultry feed, given maize’s high proportion in such feed (a major carrier of FB1), not only poses a significant threat to poultry health and causes economic losses to the poultry industry, but also seriously undermines the safety of poultry-derived food products (e.g., meat, eggs) due to persistent toxin residues, raising risks for human consumers. In chickens, FB1 exhibits low bioavailability due to poor absorption in the avian gastrointestinal tract. Studies have shown that FB1 is rarely absorbed across the chicken gastrointestinal tract [6,7], with most of the toxin remaining in the intestinal lumen rather than entering the systemic circulation. This poor absorption leads to FB1 being primarily retained within the intestinal lumen, resulting in prolonged exposure of chicken intestinal epithelial cells to locally high concentrations of the toxin. FB1 ingestion can adversely affect the gastrointestinal function of animals through multiple mechanisms such as increasing gastrointestinal heat shock proteins [37], regulating intestinal flora homeostasis [38,39], disrupting the barrier function [40,41] and altering chemokine expression [42] of intestinal epithelial cells and gut tissue. Accumulating evidence underscores the damaging effects of FB1 on vulnerable intestinal structures, and there is an urgent need for an in-depth assessment of its mechanisms of action in intestinal barrier dysfunction [43,44]. However, there is still a lack of ex vivo intestinal models for chickens that incorporate multiple cell types (e.g., enterocytes, goblet cells, intestinal stem cells), which limits comprehensive investigations into gut barrier function, host-microbe interactions, and species-specific responses to toxins or pathogens. To address this issue, we initially developed a 3D intestinal organoid model to recapitulate the complex intestinal microenvironment, and subsequently optimized it into a monolayer to simulate direct contact between intestinal epithelial cells and toxins. Using this model, we investigated the influence of FB1 exposure on intestinal barrier integrity, inflammatory responses and self-renewal ability of intestinal stem cells, aiming to elucidate the specific mechanism of FB1 tolerance in poultry.

The intestinal mucosal barrier serves a fundamental role in maintaining gut homeostasis, encompassing a physical barrier, a chemical barrier, an immune barrier and a microbial barrier [45]. The integrity of the physical barrier is commonly evaluated through the expression of tight junctions (TJs) and TEER. TJs are composed of transmembrane proteins such as Claudin1, Occludin, and ZO1. Generally, mycotoxins disrupt the barrier function of intestinal epithelial cells. Aflatoxin B1 (AFB1) at high concentration induces a time-dependent decrease in transcript levels of *CLDN3* and *OCLN* and TEER values of Caco2 cells [46,47]. Similarly, ochratoxin A (OTA) exposure significantly downregulates the mRNA expression of *CLDN3*, *CLDN4*, and *OCLN* in Caco-2 cells [46]. Contradictory evidence has been reported in animal models. AFB1 exposure does not increase gut permeability in broiler chickens [48]. Collectively, these findings highlight that mycotoxin-induced intestinal barrier dysfunction exhibits both dose-dependent and time-dependent characteristics. In the present study, exposure to FB1 did not result in decreased TEER values or abnormal expression of tight junction genes (*ZO1*, *Occludin*) in the chicken intestinal organoid monolayers, while the levels of pro-inflammatory factors (*IL6*, *TNF-α*) and anti-inflammatory factors (*IL10*) did not fluctuate significantly, suggesting that the intestinal barrier function and the inflammatory homeostasis of chickens are uniquely tolerant to FB1.

The sensitivity to FB1 exposure varies significantly among animal species. In pigs, FB1 has high bioavailability and accumulates throughout the body (liver, lungs, kidneys), whereas in chickens, the toxin is poorly absorbed and is mostly confined to the intestine. Pigs are highly susceptible to FB1, as low doses (lowest observed adverse effect level, LOAEL: 5.0 mg/kg feed or 0.2 mg/kg body weight per day) can induce pulmonary edema [49]. This sensitivity is attributed to the inhibition of ceremide synthase and the accumulation of bioactive sphingoid bases. In contrast, unlike mammals, poultry are relatively resistant to FB1, requiring substantially higher doses (LOAEL: 40 mg/kg feed or 4.7 mg/kg body weight per day) to induce intestinal damage [8]. The varying sensitivity to FB1 across animal species is likely attributed to differences in detoxification capacities. Poultry, for instance, exhibit a faster metabolic rate and higher body temperature compared to mammals [50]. Additionally, the jejunal mucosa of chicks is reported to regenerate approximately every 48 h, suggesting that rapid epithelia turnover may mitigate FB1 toxicity. Furthermore, mycotoxins such as zearalenone can influence epithelial repair and maintain homeostasis by regulating the Wnt/β-catenin signaling pathway [22]. T-2 toxin activaties the Wnt/β-catenin signaling pathway in mice kidneys and HK-2 cells, thereby promoting kidney fibrosis [51]. Similarly, mycotoxin Sporidesmin A triggers both canonical and noncanonical Wnt/β-catenin signaling [52]. Consistent with these findings, our study also showed that FB1 exposure promoted activation of the Wnt/β-catenin signaling pathway and upregulated the expression of Wnt-associated genes (*β-catenin*, *Cyclin D1* and *c-myc*) as well as proliferation-related genes (*PCNA* and *Ki67*). Thus, we propose that a higher turnover rate of intestinal epithelial cells driven by the Wnt/β-catenin signaling pathway may contribute to poultry’s resistance to FB1. However, it is also important to mention that excessive or chronic activation of this pathway can result in pathological outcomes, including the malignant transformation of the intestinal epithelium [53,54].

## 5. Conclusions

In summary, we established a chicken intestinal organoid model consisting of multiple intestinal cell types, and further constructed it into a functional chicken intestinal organoid monolayer model with epithelial integrity. In addition, we demonstrated that the chicken intestinal organoid monolayer exhibited specific tolerance to FB1 exposure. Notably, FB1 activated the Wnt/β-catenin pathway to enhance chicken intestinal stem cell-driven epithelial regeneration, resulting in increased resistance to FB1 and reduced sensitivity to the toxin. The present study reveals a new strategy to enhance tolerance to FB1 in poultry by regulating intestinal stem cell function, which provides a new perspective for the development of mycotoxin prevention and control strategies.

## Data Availability

The authors confirm that the data supporting the findings of this study are available within the article and its Supplementary Materials.

## Supplementary Materials

The following supporting information can be downloaded at: https://www.mdpi.com/article/10.3390/ani15192850/s1, Figure S1. (A) Cell viability of IPEC-J2 cells treated with different concentrations of FB1. (B–H) mRNA levels of tight junction proteins (*ZO1*, *Occludin*, and *Claudin1*), goblet cell secreted mucin (*MUC2*), and pro- and anti-inflammatory cytokines (*IL6*, *TNF-α*, and *IL10*) from FB1-treated IPEC-J2 cells at 48 h. * *p* < 0.05, ** *p* < 0.01, ns: not significant. Table S1. Primer sequences for RT-qPCR.
