# Supplementary material for: Wnt/β-Catenin Pathway Activation Confers Fumonisin B1 Tolerance in Chicken Intestinal Organoid Monolayers by Enhancing Intestinal Stem Cell Function
Source: Animals (Basel). 2025 Sep 29;15(19):2850. doi: 10.3390/ani15192850 (PMC12523766; doi:10.3390/ani15192850)
Supplement: Supplementary file 1 [file animals-15-02850-s001.zip › animals-3868067-supplementary.pdf]

Supplementary material for

Wnt/β-Catenin Pathway Activation Confers Fumonisin B1 Tolerance in Chicken Intestinal Organoid Monolayers by Enhancing Intestinal Stem Cell Function

Shuai Zhang [†], Yanan Cao [†], Yiyi Shan, Xueli Zhang, Liangxing Xia, Haifei Wang, Shenglong Wu * and Wenbin Bao *

College of Animal Science and Technology, Yangzhou University, Yangzhou 225009, China; shuai_zhang1990@163.com (S.Z.); yncao1994@163.com (Y.C.); mz120211434@stu.yzu.edu.cn (Y.S.); zhangxueli1632022@163.com (X.Z.); lxxia0830@163.com (L.X.); hyfiwang@yzu.edu.cn (H.W.)

* Correspondence: slwu@yzu.edu.cn (S.W.); wbbao@yzu.edu.cn (W.B.); Tel.: +86-13815808787 (S.W.); +86-514-87979316 (W.B.)

† These authors contributed equally to this work.

Number of pages: 3

Number of figures: 1

Number of tables: 1

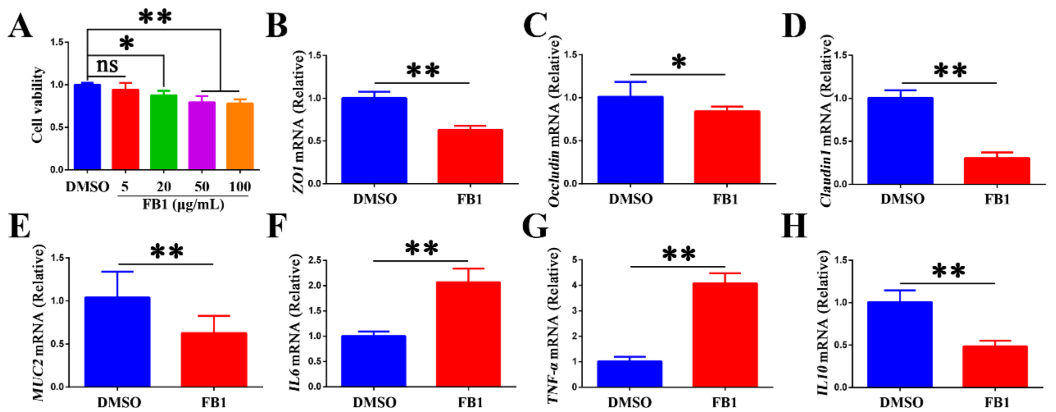

Figure S1. (A) Cell viability of IPEC-J2 cells treated with different concentration of FB1. (B-H) mRNA levels of tight junction proteins (*ZO1*, *Occludin*, and *Claudin1*), goblet cell secreted mucin (*MUC2*), and pro- and anti-inflammatory cytokines (*IL6*, *TNF-α*, and *IL10*) from FB1-treated IPEC-J2 cells at 48 h. * $p < 0.05$, ** $p < 0.01$, ns not significant.

Table S1 Primer sequences for RT-qPCR

Primers	Sequence (5′ - 3′)	Accession
Chicken-GAPDH	F: TGATGGTCCACATGGCATCC<br>R: GGGAACAGAACTGGCCTCTC	NM_204305.1
Chicken-ZO1	F: GACAGCCAACAAGGCAAGTG<br>R: TGCATCCCATCCAGGTCCTA	XM_046925214.1
Chicken-Occludin	F: ACAGCCCTCAATACCAGGATGTG<br>R: ACCATGCGCTTGATGTGGAA	NM_205128.1
Chicken-Claudin1	F: AGGTGTACGACTCGCTGCTT<br>R: GGGCATTTTTGGGGTAGCCT	NM_001013611.2
Chicken-MUC2	F: ATTGTGGTAACACCAACATTCATC<br>R: CTTTATAATGTCAGCACCAACTTCTC	XM_040673077.2
Chicken-IL6	F: CGGCAGATGGTGATAAATCC<br>R: CCCTCACGGTCTTCTCCATA	NM_204628.2
Chicken-TNF-α	F: GGCAGCTGTGGTGCAAATAA<br>R: CACAACACGGCTTCAGCATC	NM_204267.2
Chicken-IL10	F: AGGAGCAAAGCCATCAAGCA<br>R: ACCGAACGTTAAGCTGCCAT	NM_001004414.4
Chicken-Wnt3a	F: CCGGGGTTGGGTAGAAACTC<br>R: GGTCACAGCCATCAATCCCA	NM_001081696.2
Chicken-Lrp5	F: TGGCTGTAGACTGGATGGGA<br>R: ACAGCCGTTGGTCTGCATAA	NM_001012897.2
Chicken-β-catenin	F: TCTCACATCACCGTGAAGGC<br>R: TGGAGCAGACTGACAACACC	NM_205081.3
Chicken-TCF4	F: TTCGGAAGGTTCCTCCGGG<br>R: ATGCGAGTGCTTTCCCCAAC	XM_046937299.1

Chicken-Lgr5	F: TACGTCTTGCAGGAAATGGCT<br>R: GGAACCTGGCGTAGTTGGTTA	XM_046909876.1
Chicken-Cyclin D1	F: GACCCGACGAGTTACTGCAA<br>R: GAGCCACAAAAGTCTGAGCA	NM_001396513.1
Chicken-C-myc	F: ATCGACCCCTCGGTGGTCTT<br>R: GGCTGGGTATTCCACCTTGG	NM_001030952.2
Chicken-PCNA	F: AATGCGGATACGTTGGCTCT<br>R: CACCAATGTGGCTGAGGTCT	NM_204170.3
Chicken-Ki67	F: GAAGACAGTGAGCGGCTGT<br>R: TGTTTGAGGCTTAGCTGGCA	XM_040674400.2
Chicken-BMI1	F: ATCGTGCGGTACTTGGAGAC<br>R: TTTGAAAAGGCCCGGTACGA	NM_001007988.3
Chicken-SOX9	F: TCATCCTCCAGCATCAGCGA<br>R: ATGTCCACGTCTCGGAAATCG	NM_204281.2
Chicken-Lyz	F: TGAAGCGTCACGGACTTGAT<br>R: TCTCGAATTTTGCGGCACAC	NM_205281.2
